# The presence of living endometrial cells in ovarian endometriotic cyst fluid may contribute to the recurrence of endometriosis after surgical excision of endometriomas

**DOI:** 10.1186/s13048-022-01018-9

**Published:** 2022-07-30

**Authors:** Xinxin Xu, Yichen Chen, Qin Yu, Jianzhang Wang, Ping Xu, Libo Zhu, Qiong Xu, Jing Zhang, Shuling Cui, Kewen Yu, Tiantian Li, Xinyue Guo, Xinmei Zhang

**Affiliations:** 1 Department of Gynaecology, Women’s Hospital, School of Medicine, Zhejiang University, 310006 Hangzhou, P.R. China; 2 Department of Drug and Pharmacology, Ningbo Institute of Medical Science, Ningbo, 315000 P.R. China; 3 Department of Radiology, Women’s Hospital, School of Medicine, Zhejiang University, Hangzhou, 310006 P.R. China; 4 Department of Gynaecology, The Affiliated Hospital of Medical School, Ningbo University, Ningbo, 315000 P.R. China; 5 Department of Gynaecology, Ningbo Women & Children’s Hospital, Ningbo University, Ningbo, 315000 P.R. China

**Keywords:** Ovarian endometriosis, Endometriotic cyst fluid, Endometrial cells, Surgery, Recurrence

## Abstract

**Background:**

Many factors can affect the recurrence of endometriosis after surgery, however, whether endometriotic cyst fluid contributes to endometriosis recurrence after surgical excision of ovarian endometriomas remains unclear. The objective of this study was to determine the presence of endometrial cells in ovarian endometriosis cyst fluid and the potential differences between these cells and those in the cyst wall.

**Methods:**

Samples of cyst fluid (*n* = 39) and drainage fluid (*n* = 14) were collected from patients with ovarian endometriomas undergoing laparoscopic surgery. Drainage fluid from 14 patients without endometriosis was used as a control. The presence of endometrial cells in cyst fluid and drainage fluid was determined by cell culture in vitro and immunostaining. In addition, cyst fluid endometrial fragments and viscosity were analysed by transcriptome sequencing analysis and apparent diffusion coefficients, respectively. An animal model was used to confirm the ability of endometrial cells in cyst fluid to form new lesions.

**Results:**

We found endometrium-like tissues in 71.8% (28/39) of cyst fluid and 71.4% (10/14) of drainage fluid samples by histopathological examination, and the presence of endometrioid tissue in cyst fluid was related to the viscosity of the cyst fluid. The living endometrial cells in cyst fluid and drainage fluid were confirmed by cell culture in vitro and immunostaining. Moreover, the adhesion ability of endometrial fragments in cyst fluid was significantly higher than that of ectopic tissues in the cyst wall (*P* < 0.05). In addition, living endometrial cells in the cyst fluid were able to adhere and alive in the animal model.

**Conclusions:**

The existence of living endometrial cells with high adhesion ability in ovarian endometriotic cyst fluid may contribute to the recurrence of endometriosis after surgical excision of endometriomas due to cyst fluid outflow during the surgical procedure.

**Supplementary Information:**

The online version contains supplementary material available at 10.1186/s13048-022-01018-9.

## Background

Endometriosis is a chronic systemic disease characterized by the implantation and growth of endometrial-like tissue outside the uterus and affects 6-10% of reproductive-age women [1, 2]. The main manifestations of endometriosis are pain and subfertility, which greatly impact women’s physical and mental health [3, 4]. Currently, medical therapy is administered to patients who have mild symptoms and need long-term management after conservative surgery, but it is often unbearable for a long time due to the side effects of the drugs, resulting in the recurrence of symptoms after drug withdrawal [4, 5]. Surgical treatment is focused on patients with severe symptoms and pregnancy failure, but it is associated with a high rate of symptom recurrence after operation [6–8]. Recurrence is a substantial problem in the clinical treatment of endometriosis.

All types of endometriosis have a high rate of recurrence after conservative surgery [9]. For ovarian endometriosis, the recurrence rates are 9.2 and 15.4% at 3 and 5 years after surgical excision of endometriomas [10, 11]. For superficial endometriosis, the recurrence rates at 1 and 2 years following laparoscopic surgery are 4.1 and 6.7%, respectively [12]. For deep infiltrating endometriosis, a recurrence rate of 2.1-43.5% at 1-5 years is observed after surgical excision of deep nodules [13, 14]. Moreover, the reoperation rates of endometriosis at 2 and 7 years after conservative surgery are 21 and 58%, respectively [15]. Apparently, recurrence is inevitable because endometriosis is a progressive disease [6]. Although many factors, such as age, residual endometriotic lesions, pregnancy, surgical approach and postoperative drug therapy, can affect the recurrence of endometriosis after surgery, the mode and thoroughness of surgical excision of endometriotic lesions may be the key factors in the recurrence of endometriosis [5, 10, 12, 16]. Clinically, two methods are often used to treat cyst fluid before ovarian endometriotic cyst removal. One is to perform a puncture, aspiration and wash strategy before cyst removal, and the other is to directly perform suction and to rinse while separating the adhesion of the cyst. However, no matter which method is used, the overflow of ovarian endometriotic cyst fluid is inevitable during the surgical procedure. Ovarian endometriotic cyst fluid contains a variety of factors associated with the pathogenesis of endometriosis, including dead erythrocytes, reactive oxygen species (ROS), free iron and high concentrations of bilirubin and ferritin [17–20]. This implies that ovarian endometriotic cyst fluid is involved in the pathogenesis of endometriosis. Some researchers have focused on the effect of human endometriotic cyst fluid in causing endometriosis or postoperative adhesion in animal models such as mice or rabbits [21, 22]. However, it is unclear whether endometrial cells exist in ovarian endometriotic cyst fluid, thus possibly contributing to the postoperative recurrence of endometriosis.

Therefore, in this study, we aimed to determine the presence of endometrial cells in ovarian endometriotic cyst fluid and postoperative peritoneal drainage fluid and the potential differences in endometrial tissue between cyst fluid and the cyst wall (namely, ectopic endometrium). We found living endometrial cells in ovarian endometriotic cyst fluid and postoperative peritoneal drainage fluid. Moreover, the function of living endometrial cells in the cyst fluid was proved by animal experiments. Our results indicate that endometrial cells in cyst fluid caused by cyst fluid outflow during the surgical procedure may contribute to the postoperative recurrence of endometriosis.

## Results

### Correlation of ADC value and cyst fluid viscosity

**Fig. 1:**
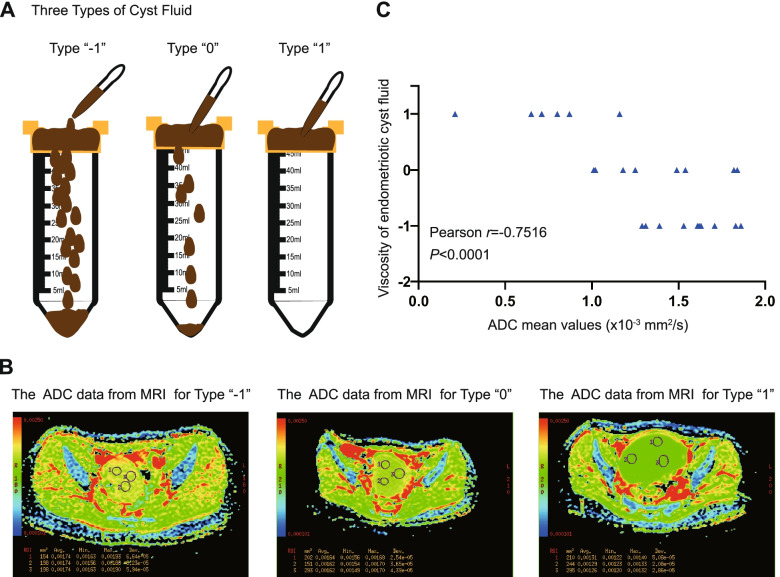
Correlation of ADC index and cyst fluid viscosity. **A** Schematic diagram of viscosity classification of endometriotic cyst fluid. **B** The ADC index of endometrioma with different cyst fluid viscosities. **C** The correlation between the ADC value and the viscosity level. The correlation was analysed using Pearson correlation. Pearson *r* = − 0.7516, *P* < 0.0001, *n* = 24

Figure 1A and B show the classification of viscosity and determination of the apparent diffusion coefficient (ADC) in endometrioma. The ADC value (× 10^− 3^ mm^2^/s) was inversely proportional to the viscosity value defined by us. The viscosity cyst fluid had a smaller ADC value (Fig. 1C). The ADC values of each patient are listed in Additional file 2.

### Presence of living endometrial cells in cyst fluid and drainage fluid

**Fig. 2:**
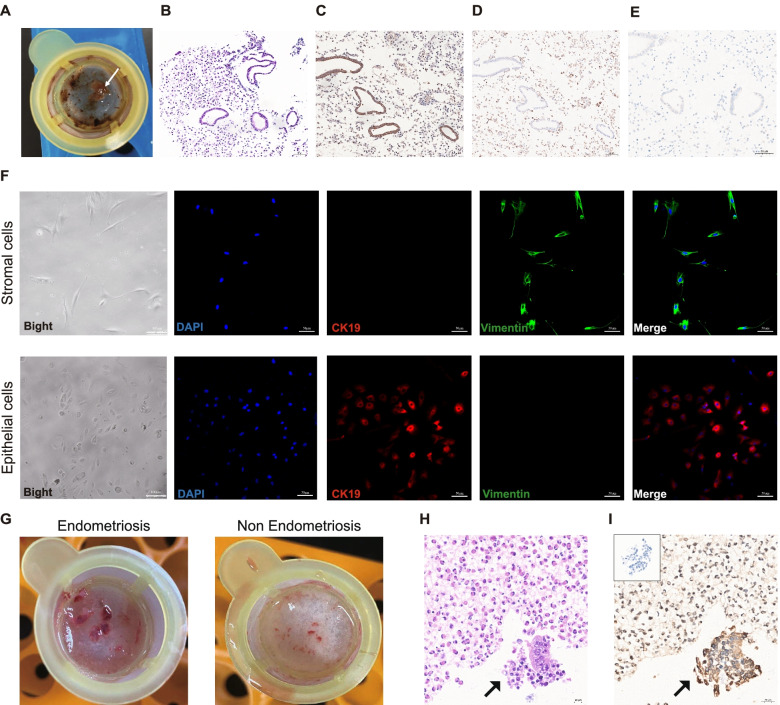
Presence of living endometrial cells in cyst fluid and drainage fluid. **A** The white arrow shows the endometrial fragments filtered out from cyst fluid. **B** H&E staining of endometrial fragments; × 200; bar = 50 μm. **C** IHC staining of cytokeratin 19 in endometrial fragments; × 200; bar = 50 μm. **D** IHC staining of CD10 in endometrial fragments; × 200; bar = 50 μm. **E** Negative control for immunohistochemistry staining in endometrial fragments; × 200; bar = 50 μm. **F** Morphology and identification of primary cultured cells from endometrial fragments. Bright field, × 100, bar = 100 μm; IF field; × 200; bar = 50 μm. Stromal cells (vimentin in green) and epithelial cells (cytokeratin19 in red). **G** Representative images of tissue in the drainage bag from endometriosis and non-endometriosis patients. **H** Representative images of H&E staining in tissue from the drainage fluid of endometriosis; × 400; bar = 20 μm. **I** Representative images of IHC staining of cytokeratin 19 in tissue from the drainage fluid of endometriosis (Negative control was in the box in the upper left corner); × 400; bar = 20 μm

We noticed that the cyst fluid graded as “-1” and “0” had endometrium-like tissues left on the filter membrane in 28 of 39 (71.8%) patients (Fig. 2A). However, in regard to grade “1” cyst fluid, with increased viscosity, noticeable fragments were not easily found with the sieve. Haematoxylin and eosin (H&E) and immunohistochemistry (IHC) staining were performed on the endometrial fragments. Representative images of H&E staining showed loose glandular and stromal structures (Fig. 2B). Positive staining of CK19 indicated a monolayer of cuboidal epithelial cells (Fig. 2C). CD10 staining showed stromal cells in the endometrial fragments (Fig. 2D). Living endometrial cells cultured in vitro were observed under a microscope. Epithelial cells and stromal cells were found and identified using immunofluorescence. Stromal cells showed positive staining of vimentin and negative staining of CK19, while epithelial cells showed positive staining of CK19 and negative staining of vimentin (Fig. 2F). In this study, endometrial fragments were found in the postoperative drainage fluid in 71.4% (10/14) of patients with ovarian endometriomas. However, there were no endometrial fragments found in the drainage fluid of the non-endometriosis group (Fig. 2G). The histology and immunohistostaining of CK19 showed the existence of epithelial cells in the fragments from drainage fluid (Fig. 2H, I). No stromal cells were detected. Taken together, these results indicated the existence of biologically active endometrial fragments in the cyst fluid and drainage fluid of endometriomas.

### Potential differences in gene expression among endometrial fragments in cyst fluid, ectopic tissue on the cyst wall and eutopic endometrium

**Fig. 3:**
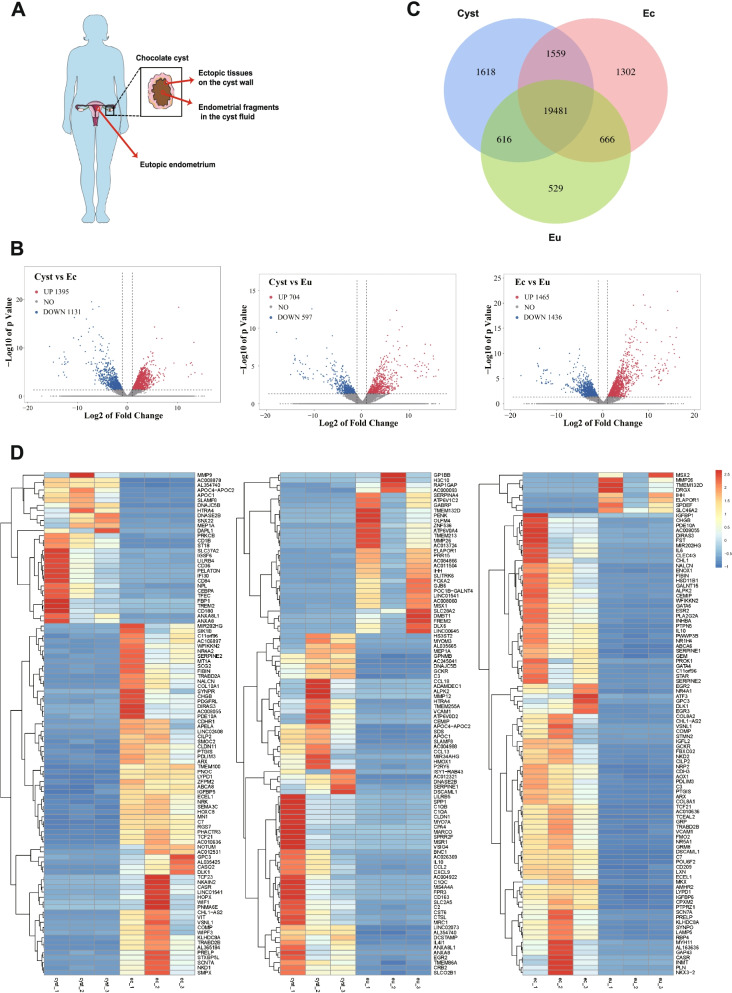
Differential gene expression within endometrial fragments (Cyst), ectopic tissue (Ec) and eutopic endometrium (Eu). **A** Schematic diagram of the collection of transcriptome sequencing samples. **B** Three volcano plots of the Cyst, Ec, and Eu groups. The red point represents upregulated genes (log2(fold change) > 1, *P* < 0.05) with statistical significance, the blue point represents downregulated genes (log2(fold change) < − 1, *P* < 0.05) with statistical significance, and the grey point in the plot represents genes with no significant differences (− 1 < log2(fold change) < 1, *P* > 0.05). **C** The Venn diagram. The blue circle represents the Cyst group. The red circle represents the Ec croup. The green circle represents the Eu group. The number in the circle indicates the number of genes uniquely expressed in each group or co-expressed within groups. **D** Heatmap of the abundance of the top 100 genes within the Cyst, Ec, and Eu groups. FPKM values are based on log2(fold change). Red and blue indicate high and low expression levels, respectively. Cyst: endometrial fragments in the cyst fluid, Ec: ectopic tissues on the cyst wall, Eu: eutopic endometrium

**Fig. 4:**
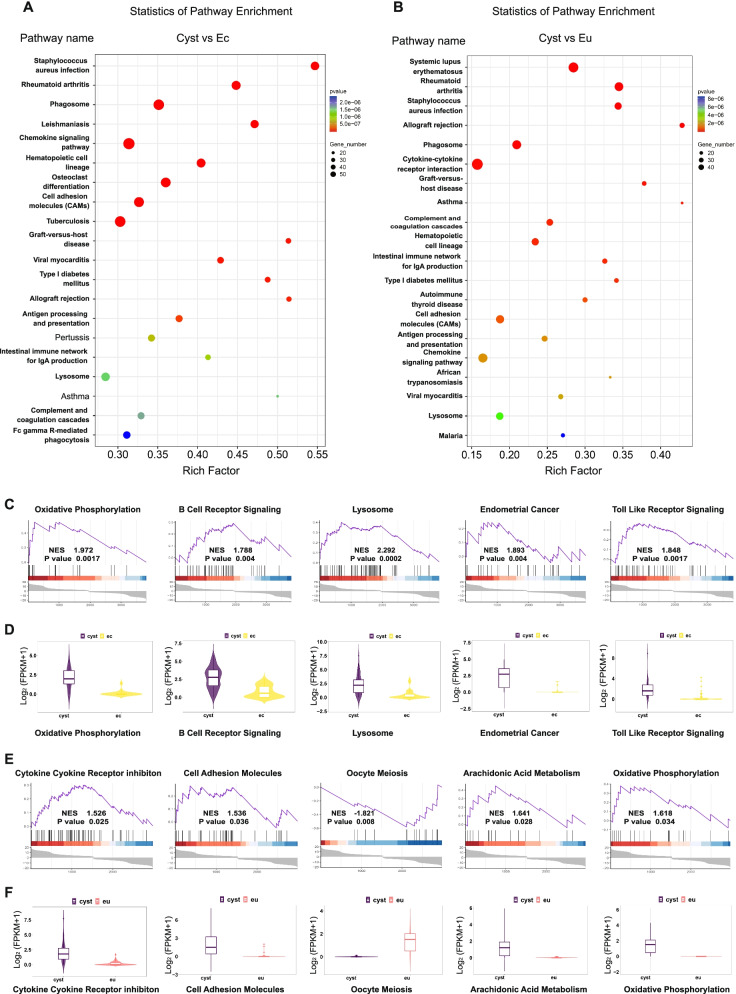
Pathway enrichment within endometrial fragments (Cyst), ectopic tissue (Ec) and eutopic endometrium (Eu). **A** Top 20 pathways enriched by KEGG between endometrial fragments in the cyst fluid (Cyst) and ectopic tissue (Ec) groups. **B** Top 20 pathways enriched by KEGG between endometrial fragments in the cyst fluid (Cyst) and eutopic tissue (Eu) groups. **C** GSEA between endometrial fragments in the cyst fluid (Cyst) and ectopic tissue (Ec) groups. **D** Violin plots of each enrichment gene set between endometrial fragments in the cyst fluid (Cyst) and ectopic tissue (Ec) groups. **E** GSEA between endometrial fragments in the cyst fluid (Cyst) and eutopic tissue (Eu) groups. **F** Violin plots of each enrichment gene set between endometrial fragments in the cyst fluid (Cyst) and eutopic tissue (Eu) groups. KEGG: Kyoto Encyclopedia of Genes and Genomes, GSEA: gene set enrichment analysis, NES: normalized enrichment score

Figure 3A illustrates the sample collection from patients. A volcano plot of the Cyst vs. Ec group showed that 1395 genes were upregulated in the Cyst group, while 1131 genes were downregulated. Moreover, in the Cyst vs. Eu group, 704 genes were upregulated and 597 genes were downregulated in the Cyst group. In the volcano plot of Ec vs. Eu, 1465 genes were upregulated and 1436 genes were downregulated in the Ec group (Fig. 3B) (most of the changes in gene expression were contained in ±5 of the log2 of the fold change). There were 19,481 genes expressed in the three types of endometrial fragments. Among these, 1618 genes were uniquely expressed in endometrial fragments from cyst fluid (Fig. 3C). The heatmaps display the differentially expressed genes between the two groups (Fig. 3D). In addition, Kyoto Encyclopedia of Genes and Genomes (KEGG) pathway analysis and gene set enrichment analysis (GSEA) of the different transcripts were conducted. The results showed that lysosomal-related genes (Fig. 4A, B), cell adhesion-related genes (Fig. 4A, B) and oxidative phosphorylation-related genes (Fig. 4C, E) were upregulated in the Cyst group compared to either the Ec or Eu group. Interestingly, endometrial carcinoma and immune-receptor-associated genes were upregulated more significantly in the Cyst group than in the Ec group (Fig. 4C). The expression of genes related to cytokine–cytokine receptors was significantly upregulated in the Cyst group compared with the Eu group (Fig. 4E), but the expression of genes related to oocyte meiosis was decreased in the Cyst group (Fig. 4E). The fragments per kilobase per million (FPKM) distribution of these pathway-related gene sets that changed significantly was analysed in a violin plot (Fig. 4D, F).

**Fig. 5:**
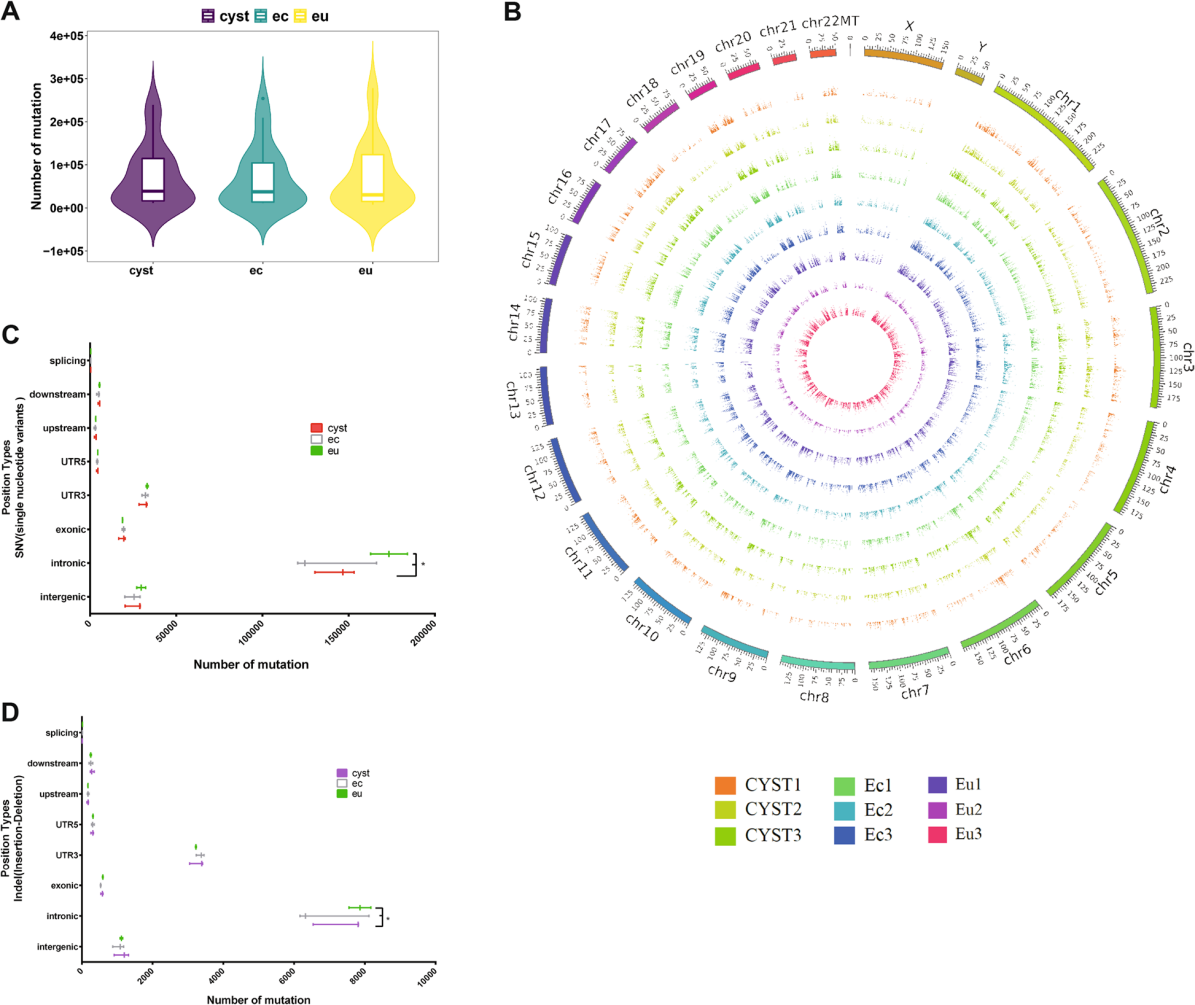
Mutation and single nucleotide variant analysis within endometrial fragments, ectopic tissue and eutopic endometrium. **A** The number and distribution of mutations in the Cyst, Ec and Eu groups. **B** Circos graph of the INDEL distribution in chromosomes in the Cyst, Ec and Eu groups. **C** The position types of SNVs in the Cyst, Ec and Eu groups. **D** The position types of INDELs in the Cyst**,** Ec and Eu groups. Eu: eutopic endometrium, Ec: ectopic lesion of the chocolate cyst, Cyst: endometrium from cyst fluid. INDELs: insertion-deletion mutations, SNVs: single nucleotide variants

We analysed the types of single nucleotide polymorphisms (SNPs) in the Cyst, Ec and Eu groups, including transition, A-G, C-T, transversion, A-C, A-T, C-G and G-T SNPs. According to the violin plot, there was a slight increase in the number of mutations in the Ec and Cyst groups compared to the Eu group, whereas there was no significant difference in the variance and frequency distribution in the three groups (Fig. 5A). We found that the distributions of insertion-deletion mutations (INDELs) in the Cyst group, Ec group and Eu group were relatively consistent in 23 pairs of chromosomes (Fig. 5B). However, both single nucleotide variants (SNVs) and INDELs tended to be intron mutations in terms of position types. Furthermore, the number of Eu group mutations at intron sites was significantly higher than that in the Ec and Cyst groups (Fig. 5C, D). Based on the similarity of the mutations, we speculate that the free endometrial fragments in the cyst fluid may come from the ectopic cyst wall.

### Increased adhesion ability in endometrial fragments from endometriotic cyst fluid

**Fig. 6:**
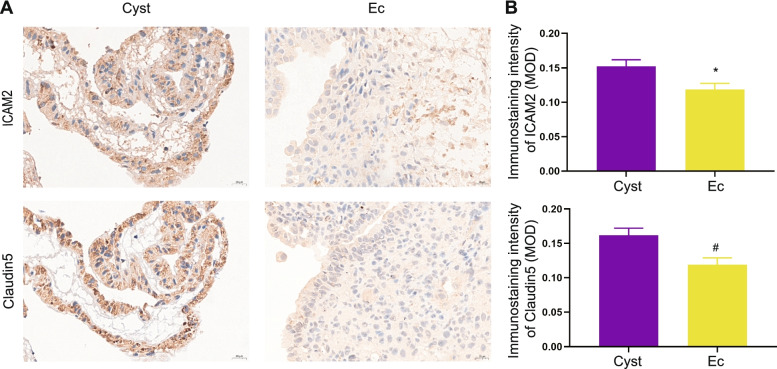
Increased expression of ICAM2 and Claudin5 in endometrial fragments from endometriotic cyst fluid. **A** Representative images of IHC staining for ICAM2 and claudin5 in endometrial fragments of cyst fluid and endometriotic lesions, × 400; bar = 20 μm. **B** The protein expression level was calculated as the mean optical density (MOD) and represented as the mean ± SEM. *P* values were determined with the Shapiro-Wilk test followed by the unpaired t-test, *n* = 8; *P = 0.0232 and #*P* = 0.0104

We detected ICAM2 and Claudin5 expression in the endometrial fragments in cyst fluid and ectopic lesions by IHC. Both the ICAM2 and Claudin5 levels were higher in the endometrial fragments in cyst fluid than in the corresponding ectopic tissue (Fig. 6A, B). Based on the above results, we hypothesize that endometrial fragments in cyst fluid have quite a strong adhesion ability.

### New lesion formation after endometriotic cyst fluid injection in vivo

**Fig. 7:**
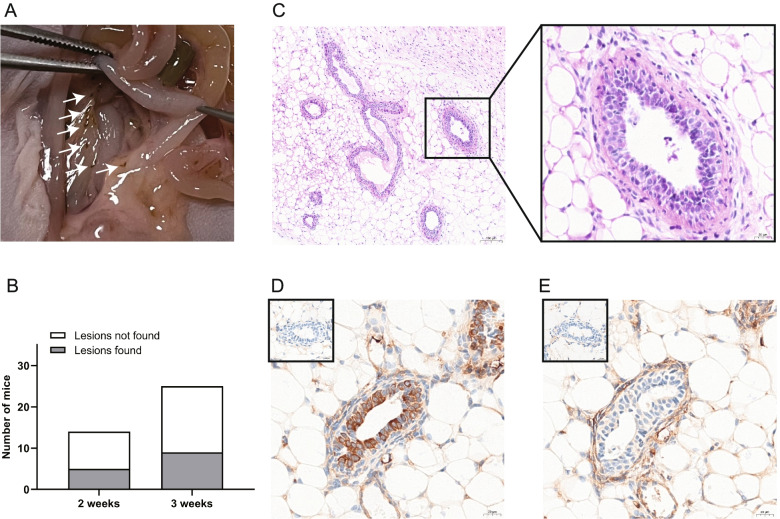
Effect of endometriotic cyst fluid injection on new lesion formation in mice. **A** The white arrows indicate the new lesions in mice after injection of endometriotic cyst fluid. **B** Number of mice with or without lesions 2 or 3 weeks after injection. **C** Representative images of H&E staining of lesions found in mice; × 100; bar = 100 μm. The black frame shows the enlarged field of view; × 400; bar = 20 μm. **D** Representative images of cytokeratin 19 staining of lesions found in mice (Negative control was in the box in the upper left corner); × 400; bar = 20 μm. **E** Representative images of CD10 staining of lesions found in mice (Negative control was in the box in the upper left corner); × 400; bar = 20 μm

To investigate the function of living endometrial cells in the cyst fluid, we injected endometriotic cyst fluid from patients into the pelvic cavity of BALB/C nude mice. Two or three weeks later, we found tiny scattered lesions wrapped by adipose tissues in the pelvic cavity of mice (Fig. 7A). Such lesions were discovered in 5 (31.25%, *n* = 16) mice at 2 weeks, and 9 (33.33%, *n* = 27) mice at 3 weeks (Fig. 7B). The endometrial structure was noticed in such lesions by histological staining (Fig. 7C). Furthermore, epithelial and stromal cells were detected by immunohistochemistry staining with cytokeratin 19 and CD10 (Fig. 7D, E). Therefore, living endometrial cells in the cyst fluid may have the ability in forming new lesions.

## Discussion

Endometriosis is an estrogen-dependent benign gynaecological disease in which endometrial tissue is presented outside the uterine cavity [23]. Although the pathogenesis of endometriosis remains unclear, the theory of retrograde menstruation is widely accepted. Retrograde menstruation is common in women; however, endometriosis was not always developed, indicating extra factors may play a role in the development of endometriosis. Accumulated research revealed that an altered peritoneal microenvironment provides a suitable condition for survival and adhesion of endometriotic cells thus contributing to the development of endometriosis [24–26].

Current therapeutic strategies for endometriosis consist of several medical therapies, surgical treatments and multidisciplinary management based on the symptoms and signs of patients. Laparoscopic surgery is widely applied in the management of endometriosis and satisfies both diagnostic and therapeutic purposes [27]. Additionally, the outflow of endometriotic cyst fluid during surgical procedures is quite common. The ‘chocolate’ fluid in the endometrioma is composed mainly of the accumulated debris derived from active implants during the menstrual cycle [28]. The complex components of the fluid in chocolate cysts can cause damage to surrounding tissues through, for instance, promoting the proliferation of endometrial cells [29], accelerating adhesion formation, aggravating fibrosis and resisting hormonal treatment [17, 18, 30]. The majority of existing studies have focused on the chemical composition in endometriotic cyst fluid [20]. Nevertheless, what we are most interested in is the potential effect of cyst fluid-derived living cells on the recurrence of endometriomas. In the current study, we observed the presence of living endometrial cells in endometriotic cyst fluid. To prevent intraoperative endometrial fragment acquisition from the cyst wall, we collected cyst fluid through needle aspiration and incisional aspiration. In addition, to avoid peeling off the inner membrane from the cyst wall, the cyst fluid was not completely drained off during sample collection. The same observation was found in the majority of the cyst fluids obtained from patients at three different hospitals, indicating that endometrial fragments with biological activity might commonly exist in cyst fluid. The tissue structure of the endometrial fragment is loose, with detached epithelial cells and a few stromal cells. The atypical morphology of endometrial cells from endometriotic cyst fluid is quite different from that of endometrial cells [31]. This phenomenon may be related to the harsh environment in endometriotic cysts. In addition, the aspiration and filtering procedures may affect cell morphology due to the small size of the free endometrial fragments. In the current study, we focused on the possible effects of cyst rupture during surgery on endometriosis recurrence. To some extent, the observation of endometrial fragments in the drainage fluid after the endometrioma operation may support the hypothesis that the intraoperative spillage of cyst content from ovarian endometriomas may be related to postoperative recurrence.

MRI is one of the common approaches for the diagnosis of endometriomas [32]. Diffusion-weighted imaging (DWI), which is based on Brownian motion, utilizes echo-planar imaging (EPI) sequences, while ADC maps can provide qualitative information. As mentioned in some previous studies, the ADC value has been suggested to be the best parameter for predicting cancer occurrence or measuring lesions before biopsy [33, 34]. Studies have shown that endometriotic cysts exhibit a lower ADC value than other functional ovarian cysts [35, 36]. The ADC mean value is also used to predict the therapeutic effect of dienogest in women with ovarian endometrioma [37]. Moreover, Guo reported that cyst fluid with higher density and viscosity has a higher adhesion score in endometriosis patients [18]. In our study, the ADC value of the endometriotic cyst in MRI was negatively correlated with the viscosity of cyst fluid, and endometrial fragments were more likely to be found in a less viscous fluid. Most likely, when the viscosity of cyst fluid increases, the environment of cyst fluid becomes more complex with the elevation of free iron [17, 30, 38] and accumulation of ROS [17, 39], which break the endometrium from the cyst wall into pieces. This correlation allowed us to predict the viscosity of the cyst fluid and to speculate whether biologically active endometrial fragments existed in the cyst fluid of endometriosis patients.

Transcriptome sequencing allowed us to further analyse the biological characteristics of the endometrial fragments in cyst fluid. The genes related to the lysosomal pathway and oxidative phosphorylation pathway in the endometrial fragments from cyst fluid were highly expressed, which is consistent with the observation that the cyst fluid is rich in many harmful substances, such as ROS and, free iron [17–20]. Various cytokines in endometriotic cyst fluid might be associated with the high expression of genes related to the arachidonic acid metabolic pathway and cytokine receptor pathway. These inflammatory cytokines might be the cause of ovarian dysfunction [40, 41]. Furthermore, genes related to oocyte maturation were expressed at lower levels in the endometrial fragments of cyst fluid, and their impact on oocytes during diffusion may lead to a loss of ovarian reserve function. Muzii et al. found that the number of follicles in ovarian tissue surrounding endometriomas was significantly decreased or even disappeared [42]. Dogan et al. reported that although the primary or secondary follicles remained at normal levels, the functional follicle was significantly reduced or even lost in ovaries with endometriomas [43]. Interestingly, the mutation sites of SNVs and INDELs were concentrated in the 3’UTR and introns. In addition, the eutopic endometrium group exhibited more mutations than the other groups. Mutations in intronic splicing enhancers or silencers have the potential to cause changes in splicing regulatory elements and can disrupt transcription regulatory motifs and non-coding RNA genes [44–48]. Notably, previous studies have reported that there are differences between the eutopic endometrium and the normal endometrium [49], which may partly be due to gene mutations in the eutopic endometrium. We conjectured that the ectopic endometrium and the endometrial fragments in cyst fluid share a similar environment. Moreover, a steady-state is of vital importance to the ectopic endometrium and the endometrial fragments in cyst fluid. Further attention will be given to the function of these intronic mutations in endometriosis in our future research.

A study showed that endometriotic cyst fluid instillation can cause adhesion in the rabbit peritoneal cavity [22]. The cause of increased adhesion ability has rarely been discussed. Previous researchers reported that soluble CD44 is highly expressed in endometriotic cyst fluid in comparison with other ovarian cysts [50]. As an integral membrane protein with multifunctional adhesion ability, CD44 plays a vital role in cancer progression, including ovarian cancer [51, 52]. In our study, we noticed that the endometrial fragments in cyst fluid had a stronger cell adhesion ability than the ectopic tissues on the wall of the cyst. Furthermore, the trauma during the surgical procedure allows living endometrial cells to attach to the abdominal cavity easily, thereby building new ectopic foci. It is often difficult to control the spread of ectopic foci and postoperative recurrence during the surgical treatment of endometriosis. The effect of increasing the amount of saline flushing or the perioperative lavage times is limited or even worse [53]. It seems that postoperative hormone therapy or preoperative intervention needs to be considered during the whole course of the treatment of endometrioma.

Some studies have examined the effect of endometriotic cyst fluid on patients in animal experiments, such as in mice and rabbits [21, 22]. Our animal experiment was conducted to further validate the effect of living cells in endometriotic cyst fluid on endometriosis recurrence. To ensure that the experimental design mimicked clinical practice as closely as possible and sufficiently tested our hypothesis, we first established an endometriosis mouse model and then performed cyst incision surgery. The components of human endometriotic cyst fluid are quite complex. After being injected into the abdominal cavity of animals, it may trigger an inflammatory reaction and the formation of adhesions, which make it difficult to identify new lesions. The application of B6-G/R [54] mice enabled us to distinguish the neo-lesions derived from the contents of the endometriotic cyst fluid in the mice. We fixed the endometrium pieces with glue to ensure that the endometriotic lesions were in a certain position after the model establishment. Subsequent new lesions were found in other parts of the abdominal cavity, indicating that the new lesions may come from the cystic fluid after our surgical intervention. Not all patients have living endometrial cells in the endometriotic cyst fluid. Similarly, not all the mice exhibited newly formed endometriotic lesions, which was consistent with clinical findings. Kennedy and his colleagues found no adhesion or endometriosis formation in mice after injection of endometriotic cyst fluid into the peritoneal cavity [21]. This is probably because the cyst fluid was taken from only one patient or transplant rejection was caused by species differences. Here, we injected endometriotic fluid from twelve patients into the pelvic cavity of BALB/C nude mice and found new lesion formation in the pelvic cavity of 33.33% of mice. Although the results of animal experiments cannot be directly extended to humans, they illustrate some problems to a certain extent.

There are several limitations to this study. Due to the particularity of the clinical samples, drainage fluid was not collected from each patient. Another limitation of our study is that there are no clinical data to prove the correlation of living endometrial cells with the recurrence rate of endometrioma in postoperative patients. We followed our patients for 3 months; however, as most of the patients were on medication after surgery, no recurrence has been observed to date, and a long-term follow-up is ongoing. During the isolation of cyst fluid, we observed the existence of immune cells, while the mixed components and the particle size in the fluid remain a difficult problem. We are now optimizing the conditions for the future isolation of immune cells from endometriotic cyst fluid. This article is an exploratory experimental study. It is indeed difficult to prove this in the human body, but experiments conducted on animals may offer some support for our hypothesis. To provide insights into and a basis for this conjecture, further human clinical research requires meticulous planning, such as increasing the number of samples and applying advanced verification experiments in further research. Furthermore, follow-up work is in progress, and, the relationship between the components in chocolate cysts and the onset and prognosis of the disease is worth exploring.

## Conclusions

Here, we found living endometrial cells in ovarian endometriotic cyst fluid and postoperative pelvic drainage fluid. The endometrial fragments in cyst fluid had a stronger adhesion ability than endometrial tissues from the cyst wall. Moreover, we found the potential ability of endometriotic cyst fluid to produce new lesions in an animal model. This evidence indicated that the outflow of cyst fluid during a surgical procedure may leave endometrial fragments in the pelvic cavity. Living endometrial cells become the hidden danger leftover in the pelvic cavity, setting the stage for recurrence. Here, we provide new insights into the recurrence of endometriosis, and we hope that more attention will be given to the surgical treatment of chocolate cysts in clinical practice, which may play a positive role in preventing or decreasing the recurrence of endometriosis.

## Materials and methods

### Sample collection of endometriotic cyst fluid, drainage fluid and endometrial tissue

A total of 39 ovarian endometriosis patients and 14 non-endometriosis controls who underwent laparoscopy between September 2020 and January 2021 were enrolled in this study after informed consent was obtained. Among them, 39 patients had ovarian endometrioma with or without endometriotic lesions in other places of the pelvis. The other 14 patients without endometriosis underwent surgical procedures for myoma, ovarian teratoma and hydrosalpinx.

Thirty-nine endometriotic cyst fluid and 14 postoperative drainage fluid samples were collected from the endometriosis patients. Fourteen postoperative drainage fluid samples were collected from non-endometriosis control patients. Ectopic tissues from the cyst wall were collected from 11 endometriosis patients and eutopic endometrium was collected from 3 endometriosis patients.

For endometriotic cyst fluid collection, the cyst fluid from 39 patients with endometriosis was randomly collected during surgery through incision aspiration or puncture aspiration (22 vs. 17). For incision aspiration, a small breach of the cyst wall was made with scissors, and the cyst fluid was collected with an aspirator connected to a 50 mL sterile syringe. Puncture aspiration was performed using a Transfix needle connected to a 50 mL sterile syringe. Approximately 5-100 mL of the endometriotic cyst fluid was aspirated into the syringe. Cyst fluid was obtained from both sides of the cysts in the patients with bilateral ovarian endometriomas. The cyst fluid was transported to the laboratory within 30 minutes on ice.

The drainage fluid was obtained from the pelvic drainage bag. Twenty-four hours after surgery, the drainage fluid (5-20 mL) was collected using a 50 mL sterile syringe from the bag and immediately transported to the laboratory in a cold chain.

The endometriotic cyst wall and eutopic endometrium were collected during the surgery and immediately transported to the laboratory in a cold chain. After being washed with cold sterile saline three times, the ectopic endometrial tissues from the cyst wall and the eutopic endometrium from the corresponding patients were immersed in liquid nitrogen for RNA extraction. The other ectopic tissues on the cyst wall were washed and immersed in 4% neutral buffered formalin for the histological experiment.

All the endometriomas were removed during the surgery. In addition, none of the patients had received sex-hormone therapy within the 6 months before surgery. Detailed information on the patients enrolled in the study and the samples used in each experiment is shown in Additional file 1.

### Classification of cyst fluid viscosity and determination of the apparent diffusion coefficient in endometriomas

The endometriotic cyst fluid was filtered through a 100 μm filter membrane, and the viscosity of the cyst fluid was classified into three types according to the difficulty of the cyst fluid passing through the filter membrane: type 1, the cyst fluid was not viscous and could directly pass through the 100 μm filter membrane, marked as “-1”; type 2, the cyst fluid had some viscosity and thereby needed to be repeatedly blown through the pipette to pass through the filter membrane, marked as “0”; type 3, the cyst fluid was very viscous and could not pass through the filter membrane even after repeated blowing, marked as “1”. It was necessary to dilute the type 3 cyst fluid more than twice until it could pass through the sieve.

Thirty-two endometriosis patients underwent MRI examination before surgery. Apparent diffusion coefficient (ADC) values were obtained from 18 patients. Fourteen patients underwent MRI in the outpatient department, and the MRI image was unavailable for analysing when they underwent the surgery. MRI scans were performed on a 1.5 Tesla MR system (Signa HDxt; GE Healthcare, Milwaukee, WI) with a phased-array abdominal coil. The patients were in a supine position and breathed freely during acquisition. The scanning range was from the inferior pubic symphysis to the renal hilum and extended beyond the dome of the cyst in patients with large masses. Diffusion-weighted imaging (DWI) was performed in axial planes at b values of 0, 800 s/mm2 (TR/TE 4600 ms/72 ms). The scanning parameters were as follows: 5 mm slice thickness, 1.2 mm gap, 256× 256 matrix, 296 mm field of view and four excitations.

ADC values were measured on ADC maps. A circular region of interest (ROI) of at least 1 cm^2^ was placed at targeted areas with the possibly lowest ADC values in the cyst components of ovarian endometrial cysts, by referring to conventional MR imaging. At least three measurements were obtained and averaged.

### Acquisition of endometrial fragments in endometriotic cyst fluid and drainage fluid

All cyst fluid or drainage fluid samples were filtered with 100 μm apertures sieves under sterile circumstances. To separate the debris and the endometrial fragments, the remnants in the 100 μm aperture sieves were washed with 1x phosphate-buffered saline (PBS) several times. Fresh, endometrial fragments were collected and washed with 1x PBS. Three endometrial fragments from cyst fluid were immersed in liquid nitrogen for RNA extraction. Ten endometrial fragments from the cyst fluid were immersed in 4% neutral buffered formalin for the histological experiment. Fifteen endometrial fragments from the cyst fluid were used for in vitro cell culture. All the endometrial fragments acquired from drainage fluid were immersed in 4% neutral buffered formalin for the histological experiment.

### In vitro culture of endometrial cells

Fragments were collected under sterile conditions and digested with type I collagenase (Biofroxx, Germany) in a shaker at 37 °C. After 45 minutes, digestion was stopped with Dulbecco’s modified Eagle medium/nutrient mixture F-12 (DMEM/F12) (1:1, Gibco, US) containing 12% foetal bovine serum. A 100 μm nylon cell strainer was used to remove the debris. Stromal cells and epithelial cells were not separated. The filtered liquid was centrifuged and resuspended in DMEM/F12 containing 12% foetal bovine serum. The cells were cultured in a humidified incubator at 37 °C with 5% CO2.

### Transcriptome sequencing and bioinformatic analysis

Total RNA of the endometrial fragments in the cyst fluid (Cyst), ectopic tissues on the cyst wall (Ec) and eutopic endometrium (Eu) was extracted using TRIzol reagent (Invitrogen). Cleaved RNA fragments were reverse-transcribed to create a cDNA library by the protocol for the mRNA Seq sample preparation kit (Illumina), with a resulting average insert size for paired-end libraries of 200 bp (± 50 bp). Samples were subjected to paired-end sequencing on an Illumina HiSeq 4000 (LC Sciences) by Lc-Bio Technologies (Hangzhou, China) Co., Ltd.

Mapped reads for each sample were assembled using String Tie. All transcriptomes were merged to reconstruct a comprehensive transcriptome using Perl scripts. Following generation of the final transcriptome, String Tie and edge R were used to estimate the expression levels of all transcripts. String Tie was used to determine the expression level of mRNAs by calculating the fragments per kilobase per million (FPKM). Differentially expressed mRNAs and genes were selected as log_2_ (fold change) > 2 or log_2_ (fold change) > − 2 and with statistical significance (*P* < 0.05). The SNP sites in coding regions were analysed based on the transcriptome level. According to the Hisat2 alignment results of each sample and the reference genome, mpileup processing was performed with SAMtools software, and the possible SNP and INDEL information of each sample was then annotated with ANOVA. Bioinformatic analysis was performed using OmicStudio tools at https://www.omicstudio.cn/tool/. We used the gene differential gene expression in the cyst, ectopic and eutopic groups as the input, and the volcano plot, heatmap, GSEA, KEGG pathway enrichment, and Venn diagram were exported from the websites. Circos graphs of INDELs were generated by Lc-Bio Technologies (Hangzhou, China) Co., Ltd.

### Immunohistochemistry staining

The endometrial fragments from cyst fluid and drainage fluid were embedded in paraffin and cut into 4 μm sections. Haematoxylin and eosin (H&E) staining was utilized to confirm the endometrial structures in the fragments. To determine the epithelial and interstitial structure, the slides were incubated with anti-cytokeratin 19 (10712-1, Proteintech, China) and anti-CD10 (18008-1, Proteintech, China) overnight. For the slides of negative control, the primary antibody was replaced by PBS. The corresponding secondary antibodies were incubated for 60 min. Slides were visualized with DAB (3,3′-diaminobenzidine), counterstained with haematoxylin and observed under a microscope.

For immunofluorescence, cells were fixed with 4% paraformaldehyde for 15 min and then permeabilized with 0.1% Triton X-100 (Sigma Aldrich) for 10 min. Unspecific binding was blocked by using 8% bovine albumin in PBS at room temperature for 1 hour. Cells were incubated overnight at 4 °C with anti-vimentin (60330-1, 1:100, Proteintech, China) and anti-cytokeratin 19 (10712-1, 1:100, Proteintech, China) antibodies. The next day, the slides were washed 3 times in PBS and incubated with goat anti-mouse antibody (Alexa Fluor® 488, ab150117, 1:500, Abcam, USA) and donkey anti-rabbit antibody (Alexa Fluor® 647, ab150075, 1:500, Abcam, USA) at room temperature for 1 hour. Nuclear DNA was labelled in blue with DAPI (ab104139, Abcam, USA). The cells were visualized under a confocal microscope (Olympus, Japan).

For the histochemical assay, paraffin-embedded slides of endometrial fragments from cyst fluid and ectopic endometrial tissue from the cyst wall were incubated with anti-ICAM2 (10121-2, 1:100, Proteintech, China) and anti-Claudin5 (GB11290, 1:100, Servicebio, China) antibodies. A semiquantitative evaluation of the immunostaining intensity was carried out using ImageJ, and the mean optical density (MOD) was used to represent the levels of protein expression. The average MOD of five different fields of a slide was regarded as the expression of the molecule.

### Animal experiment

This study was carried out in strict accordance with the National Institutes of Health Guide for the Care and Use of Laboratory Animals. Six-week-old female BALB/C nude mice were purchased from Shanghai Animal Center, Chinese Academy of Science. The mice were housed in an environment at 21 °C ± 0.5 °C under a 12 h light/dark cycle and with free access to food and water. The endometriotic cyst fluid used in this part was collected from 12 patients (The information of patients enrolled is shown in Additional file 1, sheet2). The endometriotic cyst fluid was filtered through a 100 μm filter membrane, and fresh, endometrial fragments were collected. The fragments from each patient were cut into smaller pieces and suspended in 500 μL filtered cyst fluid. The mixture from each patient was injected into the pelvic cavity of 4 mice. The mice were sacrificed after two (*n* = 16) or three (*n* = 27) weeks. The abdominal of the mouse was carefully probed and lesions were collected for histological examination.

### Statistics

Statistical analysis was performed by using GraphPad Prism 6.0 (GraphPad Software, USA). All the results are expressed as the mean ± standard error of the mean (SEM). The normality of the data was tested with the Shapiro-Wilk test. An unpaired Student’s t-test (two-tailed) was used to identify statistically significant differences between the two groups. Pearson correlation was used to analyse the correlation between the viscosity of the cyst fluid and the ADC mean value. A *P* value of < 0.05 was considered statistically significant.

## Supplementary Information

**Additional file 1.** Clinical characteristics of endometriosis and control patients in this study.

**Additional file 2.** The ADC values.

**Additional file 3: Fig. S1.** Animal model establishment and new lesion formation. **A** Schematic diagram of the animal experiment. **B** The white arrow shows endometriotic lesions in the mice after model establishment. **C** Representative image of an endometriotic lesion with ZsGreen fluorescence, × 200; bar = 50 μm. **D** The short arrow shows the lesions found in the peritoneal cavity of mice in the experimental group. **E** Representative image of a new lesion with ZsGreen fluorescence; × 200; bar = 50 μm.

## Data Availability

The data and materials supporting the study are included in this article and its additional files. The transcriptional sequencing datasets used and analysed during the current study are available from the corresponding author on reasonable request.

## Abbreviations

ROS: Reactive oxygen species
MRI: Magnetic Resonance Imaging
DWI: Diffusion-weighted imaging
ADC: Apparent diffusion coefficient
ROI: Region of interest
FPKM: Fragments Per Kilobase per Million
GSEA: Gene-set enrichment analysis
KEGG: Kyoto Encyclopedia of Genes and Genomes
CK19: Cytokeratin 19
CD10: Cluster of Differentiation 10
DAPI: 4′,6-diamidino-2-phenylindole
HE: Haematoxylin and eosin
IHC: Immunohistochemistry
ICAM2: Intercellular Adhesion Molecule 2
DAB: 3,3′-diaminobenzidine
MOD: Mean optical density
SD: Standard deviation
IF: Immunofluorescence
Eu: Eutopic tissues
Ec: Ectopic tissues
Cyst: Tissues from cyst fluid
SNPs: Single nucleotide polymorphism
INEDLs: Insertion-Deletion mutations
SNVs: Single nucleotide variants
EPI: Echo-planar imaging
UTR: Untranslated region
B6-G/R: B6/JGpt-H11em1Cin (CAG-LoxP-ZsGreen-Stop-LoxP-tdTomato)/Gpt
