# Supplementary material for: The presence of living endometrial cells in ovarian endometriotic cyst fluid may contribute to the recurrence of endometriosis after surgical excision of endometriomas
Source: J Ovarian Res. 2022 Jul 30;15:89. doi: 10.1186/s13048-022-01018-9 (PMC9338681; doi:10.1186/s13048-022-01018-9)
Supplement: Supplementary file 2 — Additional file 2. The ADC values. [file 13048_2022_1018_MOESM2_ESM.pdf]

| Number of patients | The Viscosity level of Cyst Fluid | ADC value (x$10^{-3}$ $mm^2/s$) | | | ADC mean value (x$10^{-3}$ $mm^2/s$) |
|---|---|---|---|---|---|
| 1 | -1 | 1.55 | 1.59 | 1.68 | 1.61 |
| 2 | -1 | 1.73 | 1.68 | 1.72 | 1.71 |
| 3 | -1 | 1.31 | 1.30 | 1.31 | 1.31 |
| 4 | -1 | 1.56 | 1.64 | 1.66 | 1.62 |
| 5 | -1 | 1.95 | 1.95 | 1.58 | 1.83 |
| 6 | -1 | 1.40 | 1.34 | 1.43 | 1.39 |
| 7 | -1 | 2.13 | 1.88 | 1.57 | 1.86 |
| 8 | -1 | 1.31 | 1.29 | 1.26 | 1.29 |
| 9 | -1 | 1.64 | 1.62 | 1.62 | 1.63 |
| 10 | -1 | 1.56 | 1.54 | 1.49 | 1.53 |
| 11 | 0 | 1.83 | 1.80 | 1.83 | 1.82 |
| 12 | 0 | 1.00 | 1.03 | 1.00 | 1.01 |
| 13 | 0 | 1.55 | 1.54 | 1.54 | 1.54 |
| 14 | 0 | 1.03 | 1.02 | 1.02 | 1.02 |
| 15 | 0 | 1.14 | 1.24 | 1.15 | 1.18 |
| 16 | 0 | 1.84 | 1.85 | 1.83 | 1.84 |
| 17 | 0 | 1.60 | 1.38 | 1.49 | 1.49 |
| 18 | 0 | 1.33 | 1.29 | 1.14 | 1.25 |
| 19 | 1 | 0.57 | 0.61 | 0.76 | 0.65 |
| 20 | 1 | 0.75 | 0.81 | 0.83 | 0.80 |
| 21 | 1 | 0.71 | 0.70 | 0.72 | 0.71 |
| 22 | 1 | 0.87 | 0.87 | 0.86 | 0.87 |
| 23 | 1 | 1.30 | 1.11 | 1.07 | 1.16 |
| 24 | 1 | 0.21 | 0.21 | 0.21 | 0.21 |
