# Supplementary material for: The presence of living endometrial cells in ovarian endometriotic cyst fluid may contribute to the recurrence of endometriosis after surgical excision of endometriomas
Source: J Ovarian Res. 2022 Jul 30;15:89. doi: 10.1186/s13048-022-01018-9 (PMC9338681; doi:10.1186/s13048-022-01018-9)
Supplement: Supplementary file 3 — Additional file 3: Fig. S1. Animal model establishment and new lesion formation. A Schematic diagram of the animal experiment. B The white arrow shows endometriotic lesions in the mice after model establishment. C Representative image of an endometriotic lesion with ZsGreen fluorescence, × 200; bar = 50 μm. D The short arrow shows the lesions found in the peritoneal cavity of mice in the experimental group. E Representative image of a new lesion with ZsGreen fluorescence; × 200; bar = 50 μm. [file 13048_2022_1018_MOESM3_ESM.docx]

**Effect of endometriotic cyst fluid exposure on new lesion formation in a mouse model**

To further clarify our hypothesis, we used B6-G/R mice as donors to establish an endometriosis model in C57BL/6 mice (Fig. S1A). Three weeks after implantation of endometrial tissue, six mice developed endometriosis lesions with ZsGreen fluorescence (Fig. S1B, S1C). We conducted a surgical procedure in the endometriosis mice to prove the existence of living endometrial cells and to demonstrate the ability of residual endometrial cells to form new lesions in the peritoneal cavity. In the experimental group, the cyst wall was broken down, and the cyst fluid flowed out to the peritoneal cavity. In the control group, the cyst was completely removed without outflow of the fluid. After three weeks, we found 0, 1 and 2 lesions with ZsGreen fluorescence in the abdominal cavity of three mice in the experimental group (Fig. S1D, S1E). The lesions were solid and smaller (2x1x1 mm, 3x3x3 mm, 2x2x2 mm) than the primary lesions. Moreover, the location of the newly found lesions was different from those of the established model. However, no lesions with ZsGreen fluorescence were found in three mice from the control group. Thus, during surgical excision, the outflow of endometriotic cyst fluid containing living endometrial cells may contribute to lesion reforming in an animal model.

**Fig. S1**


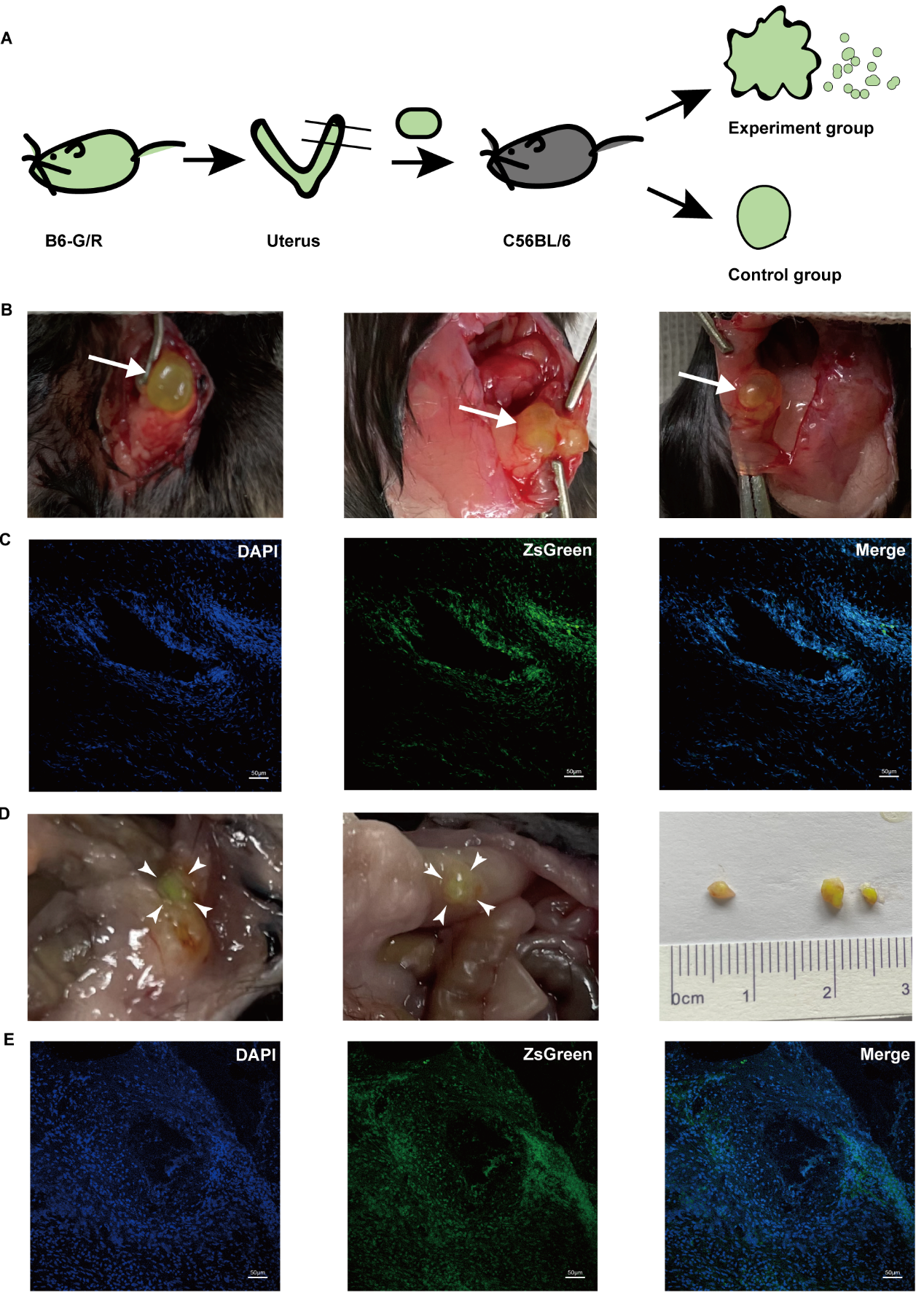


**Fig. S1** Animal model establishment and new lesion formation. **A** Schematic diagram of the animal experiment. **B** The white arrow shows endometriotic lesions in the mice after model establishment. **C** Representative image of an endometriotic lesion with ZsGreen fluorescence, x200; bar=50 µm. **D** The short arrow shows the lesions found in the peritoneal cavity of mice in the experimental group. **E** Representative image of a new lesion with ZsGreen fluorescence; x200; bar=50 µm.

**Methods:**

This study was carried out in strict accordance with the National Institutes of Health Guide for the Care and Use of Laboratory Animals. Six-week-old female B6/JGpt-H11em1Cin (CAG-LoxP-ZsGreen-Stop-LoxP-tdTomato)/Gpt (B6-G/R) mice were purchased from GemPharmatech Co., Ltd. Female C58BL/6 mice (age: 6 weeks) were purchased from Shanghai Animal Center, Chinese Academy of Science. The mice were housed in an environment at 21 °C ± 0.5 °C under a 12 h light/dark cycle and with free access to food and water. Three B6-G/R mice were used as donors. After anaesthetization with pentobarbital sodium (50 mg/Kg), a vertical incision on the abdomen was made and a distal segment of the uterine horn was removed. The endometrial tissues were immediately placed in sterile saline and trimmed for later implantation. Six C57BL/6 mice were used as receivers. After anaesthetization with pentobarbital sodium (50 mg/Kg), endometrial pieces from B6-G/R mice were stuck to each side of the peritoneum of the receiver mice using 3M Vetbond tissue adhesive (PN:1469SB). After the procedure, the peritoneum and fascia were reapproximated with 6-0 sutures in a running full-thickness stitch. The skin was closed with 4-0 sutures in a running subcuticular stitch with a buried knot.

Three weeks later, the endometriosis mice were randomly divided into two groups: the experimental group and the control group. In the experimental group, before removing the endometriotic cyst, we cut the cyst wall and allowed fluid to flow out from the cyst to the peritoneal cavity. In the control group, the complete cysts were removed carefully without rupture. The abdominal wall was closed with layer sutures. After three weeks, the mice in the different groups were sacrificed, and lesions in the peritoneal cavity were collected. The lesions of the mice were embedded in OCT (Tissue-Tek) and cut into 10 mm sections with a Leica cryostat. After staining the nuclei with DAPI, the slides were visualized under the confocal microscope (Olympus, Japan).
